# The Ability to Inoculate Purified Malaria Sporozoites Will Accelerate Vaccine and Drug Discovery

**DOI:** 10.4269/ajtmh.14-0395

**Published:** 2014-09-03

**Authors:** Michael F. Good

**Affiliations:** Institute for Glycomics, Griffith University, Gold Coast, Queensland, Australia

The ability to infect a volunteer with malaria in a controlled and safe manner promises to be of enormous benefit to research programs aimed at developing malaria vaccines^1^ or novel antimalaria drugs.^2^ By challenging an individual in early-stage trials with a defined number of parasites of a specific laboratory strain in a controlled clinical environment, it is possible to derive more meaningful data and significantly reduce trial costs, thus facilitating product development. Research presented in this issue shows that it will now be possible for trial volunteers living in both malaria-endemic and non-endemic areas.^3^

An early breakthrough occurred in 1986, when Chulay and others^4^ showed that malaria could be transmitted to naïve volunteers by mosquitoes previously fed on cultured gametocytes. In that study, the volunteers, who had never been exposed to malaria, developed a patent infection 7–11 days post-feeding.^4^ The study was a significant advance on previous approaches, in which malaria had been transmitted by mosquitoes deliberately fed on infected volunteers.^5^ However, as noted in the 1973 study,^5^ a concern relevant to both approaches was that it was impossible to determine the number of sporozoites that were injected by the mosquitoes. It was not technically possible at that time to develop a purified population of sporozoites that could be injected in a reproducible manner by syringe. This challenge lay dormant for some time, but in 2002, scientists at Sanaria commenced a major project to develop a technology to mechanically prepare sporozoites from infected mosquitoes. This technology would enable the safe infection of volunteers with defined numbers of organisms and could also be used to develop an irradiated whole-parasite vaccine (reviewed in ref.^6^). Now, after more than a decade of research, there have been three recent reports of individuals developing malaria post-inoculation of aseptic, purified, cryopreserved *Plasmodium falciparum* sporozoites.^3^,^7^,^8^ The latest report, in this issue,^3^ describes the first deliberate inoculation of individuals from a malaria-endemic area.

In the first study (involving healthy Dutch volunteers),^7^ it was shown that purified aseptic sporozoites could be administered to volunteers with a safety profile similar to mosquito bite challenge. Adverse events were attributable only to malaria and not the inoculation *per se*. Volunteers (six per group) received 2,500, 10,000, or 25,000 sporozoites injected intradermally. Of 18 recipients, 15 recipients developed a blood-stage malaria infection. In the next study,^8^ 14 of 18 healthy malaria-naïve volunteers living in the United Kingdom developed malaria after intradermal or intramuscular inoculation of sporozoites. In the latest study from Tanzania, 21 of 23 adult volunteers who had previously experienced malaria were infected after intradermal inoculation of 10,000 or 25,000 sporozoites.^3^ Infections were confirmed by microscopy and polymerase chain reaction (PCR), and heterozygosity for α-thalassemia (highly prevalent in that area) did not affect the infectivity rates.

However, these infectivity studies raise some important issues that may need to be considered to optimize this approach. Across all three studies, only 84% of individuals became infected after inoculation with up to 25,000 sporozoites (although if intramuscular inoculation is excluded, this percentage increases slightly). Why is the infectivity not 100%? By comparison, more than 95% of volunteers routinely became infected after the bites of five infected mosquitoes,^9^,^10^ although the mean number of sporozoites inoculated per mosquito may be as low as 120.^11^,^12^ Thus, sporozoites delivered by mosquito inoculation would seem to be significantly more infective than cryopreserved sporozoites delivered by syringe. It seems likely that this finding is because of the small volumes that mosquitoes inject that prevent degradation of organisms at the center of a large bolus injection, their method of inoculation, and the enhanced viability of fresh compared with cryopreserved organisms. If these differences between mosquito and syringe inoculation could be partially rectified, it is likely that significantly enhanced infectivity will be observed after syringe inoculation and thus, increase the use of this approach.

Some important lessons to address these issues can be gleaned from sporozoite infectivity studies in rodents. Ploemen and others^13^ showed that infectivity rates of *P. yoelii* sporozoites in mice after syringe inoculation decreased as the inoculation volume increased. The study by Shekalaghe and others^3^ lends support to this finding for *P. falciparum* by comparing the pre-patent period in individuals who received sporozoites in a volume of 50 μL with those who received sporozoites in a volume of only 10 μL. Although it may be difficult to reduce volumes for intradermal inoculation much farther, it would seem appropriate to consider intravenous inoculation as an alternative strategy. For example, the infectivity of *P. yoelii* sporozoites increases by more than 20-fold when delivered through the intravenous route compared with the intradermal route.^7^ Furthermore, there are no apparent safety considerations to administering sporozoites by the intravenous route.^10^

Sporozoite viability after cryopreservation is likely to be another factor affecting infectivity. Using the rodent model of *P. yoelii*, the infectivity of cryopreserved sporozoites was reduced by more than a factor of seven compared with fresh sporozoites when both were injected intravenously.^7^ If parasite viability could be increased, then the operational constraints around production of organisms at scale would be greatly diminished.

However, the technological significance of these developments to date cannot be overstated. They will allow routine challenge of individuals receiving various experimental vaccines or drugs to determine efficacy. Furthermore, they will allow for accelerated development of an irradiated sporozoite vaccine. Until recently, the most significant level of induced immunological protection from malaria was observed in volunteers exposed to the bites of over 1,000 irradiated infected mosquitoes. From the aggregates of different studies, over 90% of immunized volunteers could be protected from malaria. These data were both extremely encouraging but also frustrating in that irradiated mosquitoes could never be considered as a vaccine. However, data published in 2011 and 2013^9,10^ showed that irradiated cryopreserved sporozoites could induce immunity in a dose-dependent manner. These early encouraging vaccine results have only been possible as a result of the advances in gametocyte culture, entomological husbandry, sporozoite preparation, and cryobiology. Thus, the developments reported here and over the last 3 years may represent a watershed moment in malaria research.
